# Blindness Following Hæmorrhage

**Published:** 1885-12

**Authors:** Boerne Bettman

**Affiliations:** 113 Adams street


### BLINDNESS FOLLOWING HEMORRHAGE.

*By* BOERNE BETTMAN, M. D.

(Read before the Chicago Society of Ophthalmology and Otology.)

Cases of amaurosis following hemorrhage, although not infrequent, are, owing to the darkness enshrouding their pathology, full of interest. The two cases which I will report to the Society this evening offer certain symptoms which point

---

apparently to a common cause, namely, intracranial hemorrhage with subsequent alterations of the optic nerves.

The clinical history of the first case was extracted from the books of the Eye Clinic of Heidelberg, during my assistantship. It reads as follows:

Margaretha Feth: Aet 26, was admitted into the clinic, 1879. Patient is of healthy parentage, her mother is a hale and hearty woman of 79 years. Her father, who died three years ago of heart disease, reached the same age. Up to her fifth year she had repeated attacks of convulsions; several brothers and sisters, seven in all, died a few months after birth from the effects of similar spasms. Miss F. was otherwise a healthy, well-nourished child. Three years ago, while descending the cellar stairs, she slipped, broke and dislocated her left femur. After fifty-five days' confinement in the hospital, under the care of Prof. Nussbaum, she was discharged with a shortened limb.

On the 17th of May, 1879, while at home, she suddenly experienced a severe pain in the left side of her head, accompanied by dizziness and severe spells of vomiting. A physician was hastily summoned, he prescribed a number of powders which she could not retain in the stomach. She was unable to sleep that night; the headache continued during the next day and night, also the vomiting at frequent intervals. Her menstrual flow, which lasted from the 13th to the 18th, was not as profuse as on former occasions. On the 21st, whilst in bed, another sudden attack of dizziness resulted in unconsciousness. In this condition she remained two days. During the morning of the 22d her mother observed a large quantity of dark blood well from her mouth. The hemorrhage, according to the parent's statement, lasted fully ten minutes. The blood was not coughed up, but simply flowed from the mouth as a dark, foamy substance, mixed with a greenish mass, undoubtedly secretions from the stomach. She remained unconscious or asleep until the next morning; on awakening, all was dark around her. She was unable to see her mother standing before her with a glass of milk in her hand. Quite good sight returned to the right eye during the course of the day. A few days later, while on her way to Mannheim, dizziness again supervened, and she fell unconscious to the ground. In this state she was removed to the hospital. She regained her senses two days later. With the right eye she could barely distinguish the faces of friends close to her bed. Vision gradually diminished. On the day of admission into the clinic there was complete amaurosis of both eyes. The pupils were dilated ad maximum, the irides did not respond to light. An ophthalmoscopic examination showed decided atrophy of both discs, with thin, thread-like vessels, on both sides of which were seen broad opaque strips (perivasculitis) extending towards the periphery.

The patient remained in the hospital a long time; treatment of all varieties proved useless. Before entering the house she had taken large doses of iodide of potash, and received numerous injections of strychnine.

February 18, 1885. Dr. Hessert kindly sent me for examination a little patient, George Dohl, aged 3 years. The parents informed me that the child had fallen ten days previously against the edge of a chair, striking with the upper teeth. The boy sustained no injury except a loosening of the upper incisors. The gum bled profusely, the blood trickling down between the teeth. After all house remedies had been tried, and failed to still the hemorrhage, Dr. Hessert was called in. He succeeded in arresting the bleeding on the third day, by making digital compression, which the parents continued some time in accordance with his instructions. The following, the fourth day, was marked by long-continued, violent convulsions, lasting fully forty-eight hours. Blindness in both eyes was noticed during an interim of the spasms. The boy presented the following appearance when brought to my office: Pale, weak-looking child, pupils of both eyes dilated, reaction to light good and quite prompt. Media of both eyes clear. Papillæ very anæmic, almost perfectly white. Caliber of arteries diminished, veins somewhat dilated. Vision, O.

The child was too young to permit me to test the sensibility of its retinæ to light. My prognosis was unfavorable, still I took particular pains to call the parents' attention to one favorable feature of the case, namely, the prompt reaction of the irides, which I interpreted as indicative of a partial restoration of vision. Nourishing food, fresh air and other tonics were prescribed. Stimulants of all kinds were prohibited. I subsequently learned from Dr. Hessert that the vision had improved quite materially, sufficient to enable the child to roam about the house without danger of falling over or knocking against the various pieces of furniture. He could also distinguish smaller objects, as for instance, a knife or key, when thrown upon the floor. The child unfortunately succumbed to a second attack of convulsions, occurring eight weeks after the first.

The clinical histories of these cases appear so clear that but little difficulty will be experienced in assigning them to that category of cases mentioned in the introductory remarks. Several theories have been advanced to explain the relationship between these hemorrhages and amaurosis. The oldest and by far the most plausible theory is the one apparently adhered to by that critical observer, Alfred von Graefe. He relates two cases of sudden blindness following hæmatemesis.

After a thorough analysis he expresses his inabiliiy to attribute the amaurosis to any direct cause, but mentions later on (in another paper) that in all probabilities irritative changes in the optic nerve, behind the eye-ball, may be regarded as the exciting influence.*

The clinical history of his first case, that of Charles R., a farmer, aged 43 years, shows that a severe spell of vomiting (one-half quart blood), which had been preceded by eight to ten similar attacks, was followed by severe palpitation of the heart and continuous headache. On the evening of the second day following this severe hematemesis, and when he had recovered from the weakness induced by the loss of blood, he noticed a cloud was before his eyes, which developed into complete blindness six hours later. Since then his condition has undergone no material change.

Graefe attributed the haematemesis to an ulcer of the stomach.

In the second case a hemorrhage from stomach and bowels was followed two days later by dimness of sight, preceded by severe headache. The obscuration of sight increased gradually, terminating in amaurosis by the end of the second day. Graefe also states that on two rapidly following occasions, occurring soon after power of sight had been annulled, the left hand became partially paralyzed, the mouth was distorted, enunciation became difficult and indistinct.

Samelsohn† mentions two cases of amaurosis, one one-sided, which can be clearly traced to a peripheric lesion.

In two other cases recorded by him the loss of vision cannot be accounted for in the above manner, neither can it be ascribed to a central cause, the common factor of both hæ

---

*Graefe's Arch. Vol. xii., p. 149.

†Gr. Archiv. Vol. xviii., p. 225.

matemesis and amaurosis. He therefore finds refuge in another theory which he describes at great length. He is of the opinion that the pressure exerted by the blood in the brain is counterbalanced by that of the lymph column. A diminution in quantity of one fluid will be followed by an excess of the other. The result of this deficiency on the part of one and a superabundance of the other, will result in imperfect nutrition, pressure and inflammation. Thus it is supposed that an excessive hemorrhage from the stomach or rectum will deplete the system to a certain extent, and produce a cerebral anaemia. As the blood leaves the brain the lymph rushes in to produce the dire catastrophy. Dr. Samelsohn supports this hypothesis on experiments made by H. Gaethgans. This observer found that a congestion of the brain is followed by increased flow of lymph from that organ. The above explanation may be adequate to account for amaurosis of both eyes following hemorrhage, but certainly is not tenable when applied to one-sided amaurosis.

Finkensteiner's* case developed complete and gradual amaurosis following extensive gastric hemorrhage induced by ulceration of the stomach. The author hints at a possible cerebral connection between the necrotic process of the mucous membrane and the disturbance of the visual tract.

Another method (which was formerly regarded with favor) of interpreting the amaurosis, was to ascribe it to general anemia. Were this so, the archives of medicine in the Pre-Esmarck period would have been replete with instances of this kind. The most striking proof of the inconsistency of such a theory is one-sided amaurosis and the continuance of blindness in cases where the hemorrhage produced little if any anaemia. No doubt many factors come into play, and are capable of

*Gr. Archiv. Vol. viii., p. 209.

producing amaurosis, as a resultant of hemorrhage. But it appears to me that one and an almost constant occurrence in connection with the subject in question, has not received the attention it deserves, in fact it is hardly alluded to in the papers relating to this affection. It is a well-known fact that hemorrhage in all parts of the body frequently occur in debilitated subjects; that, owing either to some change in the walls of the blood vessels, or some anomaly of the circulation, hemorrhages, either by diapadasis or rhexis, are prone to occur in such individuals. I have frequently observed extravasations in the retina, in cases of pernicious anæmia,* and Litten† has recorded many such occurrences in the cases of anæmic individuals generally.

*Exertions* of all kinds naturally form an incentive to rupture of weakened blood-vessels, even those in a normal condition frequently give way, as the result of either increased or diminished pressure of the blood column. How often do we observe subconjunctional ecchymosis directly attributable to excessive spells of coughing or vomiting. Numerous cases have been reported of hemorrhages into the retinæ‡ vitreous, caused by bodily strain.

In our own cases severe attacks of vomiting and spasms, lasting in one instance several days, in the other, forty-eight hours, were undoubtedly the direct cause of the amaurosis. The sudden pain in the head experienced by the first case, followed on two occasions by vomiting, dizziness and unconsciousness, point to an intracranial lesion. In Samelsohn's cases we note that both patients complain of headache following hæmatemesis. Graefe also calls especial attention to this

*Bettman. The Condition of the Eyes in Fatal Anæmia.

†Archives Ophth. Vol. xi, p. 1.

‡Berl. kl. Wochenschr., 2d, 20 W. 21, 1877.

symptom, and mentions in this connection two slight attacks of paralysis.

The above would tend to demonstrate that although hemorrhage from stomach and the gums may be followed by amaurosis, the bleeding from these parts should not be considered as the direct cause of the blindness, but rather as a symptom concurrent with intracranial changes bearing directly upon the optic tract: hemorrhage.

113 Adams street.

